# *RP2-*Associated X-linked Retinopathy: Clinical Findings, Molecular Genetics, and Natural History in a Large Cohort of Female Carriers

**DOI:** 10.1016/j.ajo.2023.11.005

**Published:** 2024-05

**Authors:** Michalis Georgiou, Anthony G. Robson, Sami H. Uwaydat, Marco H. Ji, Ahmed F. Shakarchi, Nikolas Pontikos, Omar A. Mahroo, Michael E. Cheetham, Andrew R. Webster, Alison J. Hardcastle, Michel Michaelides

**Affiliations:** 1 From the Moorfields Eye Hospital (M.G., A.G.R., N.P., O.A.M., A.R.W., M.M.), London, United Kingdeom; 2 University College London Institute of Ophthalmology (M.G., A.G.R., N.P., O.A.M., M.E.C., A.R.W., A.J.H., M.M.), University College London, London, United Kingdom; 3 Jones Eye Institute (M.G., S.H.U., M.H.J., A.F.S.), University of Arkansas for Medical Sciences, Little Rock, Arkansas, USA

## Abstract

**Purpose:**

*RP2*-associated retinopathy typically causes severe early onset retinitis pigmentosa (RP) in affected males. However, there is a scarcity of reports describing the clinical phenotype of female carriers. We tested the hypothesis that *RP2* variants manifest in female carriers with a range of functional and anatomic characteristics.

**Design:**

Retrospective case series.

**Methods:**

Females with disease-causing variants in *RP2* were identified from investigation of pedigrees affected by *RP2* retinopathy. All case notes and results of molecular genetic testing, retinal imaging (fundus autofluorescence imaging, optical coherence tomography (OCT)), and electrophysiology were reviewed.

**Results:**

Forty pedigrees were investigated. Twenty-nine pedigrees had obligate carriers or molecularly confirmed female members with recorded relevant history and/or examination. For 8 pedigrees, data were available only from history, with patients reporting affected female relatives with RP in 4 cases and unaffected female relatives in the other 4 cases. Twenty-seven females from 21 pedigrees were examined by a retinal genetics specialist. Twenty-three patients (85%) reported no complaints and had normal vision and 4 patients had RP-associated complaints (15%). Eight patients had normal fundus examination (30%), 10 had a tapetal-like reflex (TLR; 37%), 5 had scattered peripheral pigmentation (19%), and the 4 symptomatic patients had fundus findings compatible with RP (15%). All asymptomatic patients with normal fundus, TLR, or asymptomatic pigmentary changes had a continuous ellipsoid zone on OCT when available. The electroretinograms revealed mild to severe photoreceptor dysfunction in 9 of 11 subjects, often asymmetrical, including 5 with pattern electroretinogram evidence of symmetrical (n = 4) or unilateral (n = 1 subject) macular dysfunction.

**Conclusions:**

Most carriers were asymptomatic, exhibiting subclinical characteristics such as TLR and pigmentary changes. However, female carriers of *RP2* variants can manifest RP. Family history of affected females with RP does not exclude X-linked disease. The phenotypic spectrum as described herein has prognostic and counselling implications for *RP2* carriers and patients.

## Highlights

• Deep phenotyping of the functional and anatomical characteristics of female carriers of RP2 variants, in a large cohort, identified variable severity of disease
• Female carriers can be affected with retinitis pigmentosa, but most carriers are asymptomatic

Retinitis pigmentosa (RP) can be inherited in an autosomal dominant, autosomal recessive, or X-linked (XLRP) pattern, exhibiting great phenotypic and genotypic variability.^1^, ^2^, ^3^ XLRP is a severe form of RP, with most affected males presenting with early-onset vision loss (<10 years of age), nyctalopia, nystagmus, severely abnormal or undetectable electroretinogram (ERG), and progression to legal blindness by the 3rd to 4th decade of life.^4^, ^5^, ^6^ *RPGR* and *RP2* disease-causing variants are the commonest causes of XLRP, accounting for 80% to 90% of cases.^1^ A tapetal-like reflex (TLR) can be observed both in patients and carriers with *RPGR*- and *RP2*-XLRP.^7^ Carriers of XLRP usually have mildly or moderately reduced visual function but rarely became legally blind,^8^ and it has been reported that patients with only a TLR at presentation have a better prognosis to retain visual function than those with peripheral retinal pigmentation.^9^ However, those observations were made in genetically heterogeneous groups of patients.

*RP2* (MIM 312600) is located on Xp11.23 and the encoded protein has an *N*-terminal domain with a beta helix structure similar to cofactor C, which is involved in β-tubulin folding, whereas the C-terminal domain is a ferredoxin-like alpha/beta domain.^10^^,^^11^ *RP2* disease-causing variants are responsible for 5% to 20% of cases of XLRP.^10^^,^^12^, ^13^, ^14^, ^15^, ^16^ Most male patients present with early-onset severe retinal degeneration, with early macular involvement and complete loss of the foveal photoreceptor layer by the third decade of life.^17^ Differential diagnosis of *RP2-* or *RPGR-*XLRP is challenging because no ocular measurement is genotype-specific.^4^^,^^5^ *RP2* encodes a GTPase-activating protein (GAP) for the small GTPase ARL3, and has a role in trafficking lipidated proteins in the retina to the outer segment of photoreceptors.^18^^,^^19^ Using retinal pigment epithelium (RPE) and 3-dimensional retinal organoids differentiated from patient-derived induced pluripotent stem cells (iPSCs) with an *RP2* premature stop variant, read-through drugs and AAV gene therapy rescued the cellular phenotype, supporting the feasibility of a clinical trial in patients.^20^^,^^21^ The severity and the natural history of the disease for *RP2* carriers has not been studied in depth; these data will be of value to advise patients on prognosis, as well for consideration of future gene augmentation strategies.

The current study hereby provides a detailed characterization of the clinical phenotype, molecular basis, and natural history of a large series of female carriers with *RP2* variants.

## Methods

### Subject Identification and Assessment

Females with disease-causing variants in *RP2* were identified from investigation of pedigrees affected by *RP2* retinopathy from Moorfields Eye Hospital (London, UK) and University of Arkansas Medical Science (Little Rock, Arkansas, USA) retinal genetics clinics. This retrospective study adhered to the tenets of the Declaration of Helsinki and was approved by the local ethics committees. The subjects were either obligate carriers or molecularly confirmed.

### Clinical Notes

Clinical data extracted included age of onset, visual acuity, slit-lamp biomicroscopy, and fundoscopy findings. Symptoms at presentation were also recorded. All available data were reviewed, including the findings at the last available follow-up.

### Best-Corrected Visual Acuity and Clinical Severity Grading

Best*-*corrected visual acuity (BCVA) was assessed monocularly with a Snellen chart and converted to logarithmic minimum angle of resolution (logMAR). Jayasundera and associates^16^ have described an approach to subdivide *RP2*-XLRP patients into mild, less severe, and severe categories. Patients with relatively late onset severe macular dysfunction were considered less severe. BCVA with different cutoffs for different age ranges was used as a subjective surrogate for macular function. We adopted and adapted the same clinical severity grading criteria into logMAR and applied it for the best seeing eye (Supplemental Table 1), as we previously did for affected males with *RP2* variants.^17^

In addition, BCVA of the best-seeing eye was used to categorize patients into 1 of 4 groups based on the World Health Organization (WHO) visual impairment criteria, that defines a person with no or mild visual impairment when VA is ≤0.48 logMAR, moderate impairment when VA is 0.48 to 1 logMAR, severe if 1 to 1.3 logMAR, and blindness if it is >1.3 logMAR (Supplemental Table 1). Low vision corresponds to patients with moderate and severe impairment. Counting fingers vision was given a value of logMAR 1.98 and hand motion, logMAR 2.28, light perception and no light perception were specified as logMAR 2.7 and 3, respectively.^22^ The BCVA classification criteria are summarized in Supplemental Table 1.

### Electrophysiological Testing

Pattern electroretinogram (PERG) and full-field ERG testing was performed incorporating the standards of the International Society for Clinical Electrophysiology of Vision (ISCEV).^23^^,^^24^ Pattern ERG P50 was used as an objective measure of macular function and the full-field ERG used to assess generalized rod and cone system function. ERG data were compared with a reference range from a group of healthy subjects (age range 10-79 years).^25^^,^^26^ The amplitudes of the main full-field ERG components were plotted as a percentage of the age-matched lower limit of normal, including the dark adapted (DA) 10 ERG a- and b-waves, and the light-adapted (LA) 3 single flash ERG b-wave and the LA 3 30Hz ERG. To address non-Gaussian distribution within the control group, the limits were defined as the lowest value in the control group minus 5% of the reference range (maximum minus minimum values) for amplitudes or the maximum plus 5% of the reference range for peak times.^27^^,^^28^

### Fundus Autofluorescence

Fundus autofluorescence (FAF) images were obtained using short-wavelength excitation (488 nm) or medium wavelength (532 nm) and a scanning laser ophthalmoscope according to previously described methods.^29^ Images were reviewed by 1 grader (M.G.) and qualitatively graded.

### OCT

Horizontal scans acquired using the Heidelberg Spectralis OCT (Heidelberg Engineering, Heidelberg, Germany) were chosen for evaluating the integrity of the ellipsoid zone.

### Statistical Analysis

Statistical analysis was carried out using SPSS Statistics for Windows software (v 22.0; IBM Corp, Armonk, New York, USA). Significance for all statistical tests was set at *P* < .05. The Shapiro-Wilk test was used to test for normality for all variables.

## Results

### Subject Identification

Forty pedigrees were identified. Thirty-eight pedigrees had affected males, and the genetics of those pedigrees were presented in detail in our study characterizing the phenotype of affected males.^17^ The additional 2 pedigrees were identified after molecular confirmation of females with abnormal fundus (TLR and pigmentary changes). No eligible females were identified from 11 pedigrees because of a lack of segregation in female members (n = 4), de novo mutation (n = 1) or female members were not examined, or relevant history was not recorded in the medical record (n = 6). Twenty-nine pedigrees had obligate carriers or molecularly confirmed female members.

### Retinal Phenotype and Presentation

**FIGURE 1:**
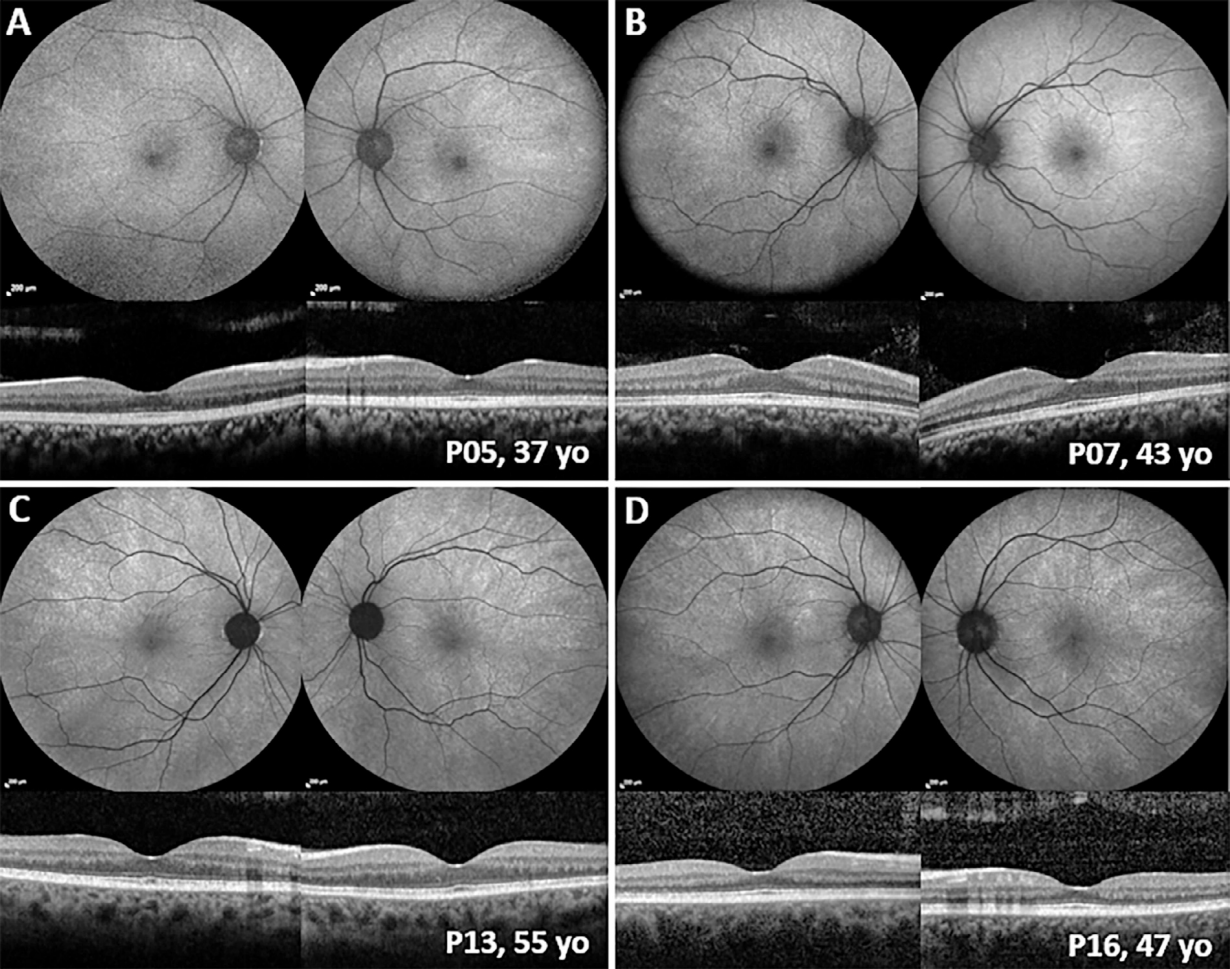
Retinal imaging of asymptomatic *RP2* carriers. Fundus autofluorescence and optical coherence tomography scans of 4 patients with tapetal-like reflex. In all 4 patients, the radial pattern changes were obvious on fundus autofluorescence. Optical coherence tomography showed preserved retinal layering with hyperreflectivity of the ellipsoid zone. Retinal changes were symmetrical between eyes. P = patient; yo = years old.

**FIGURE 2:**
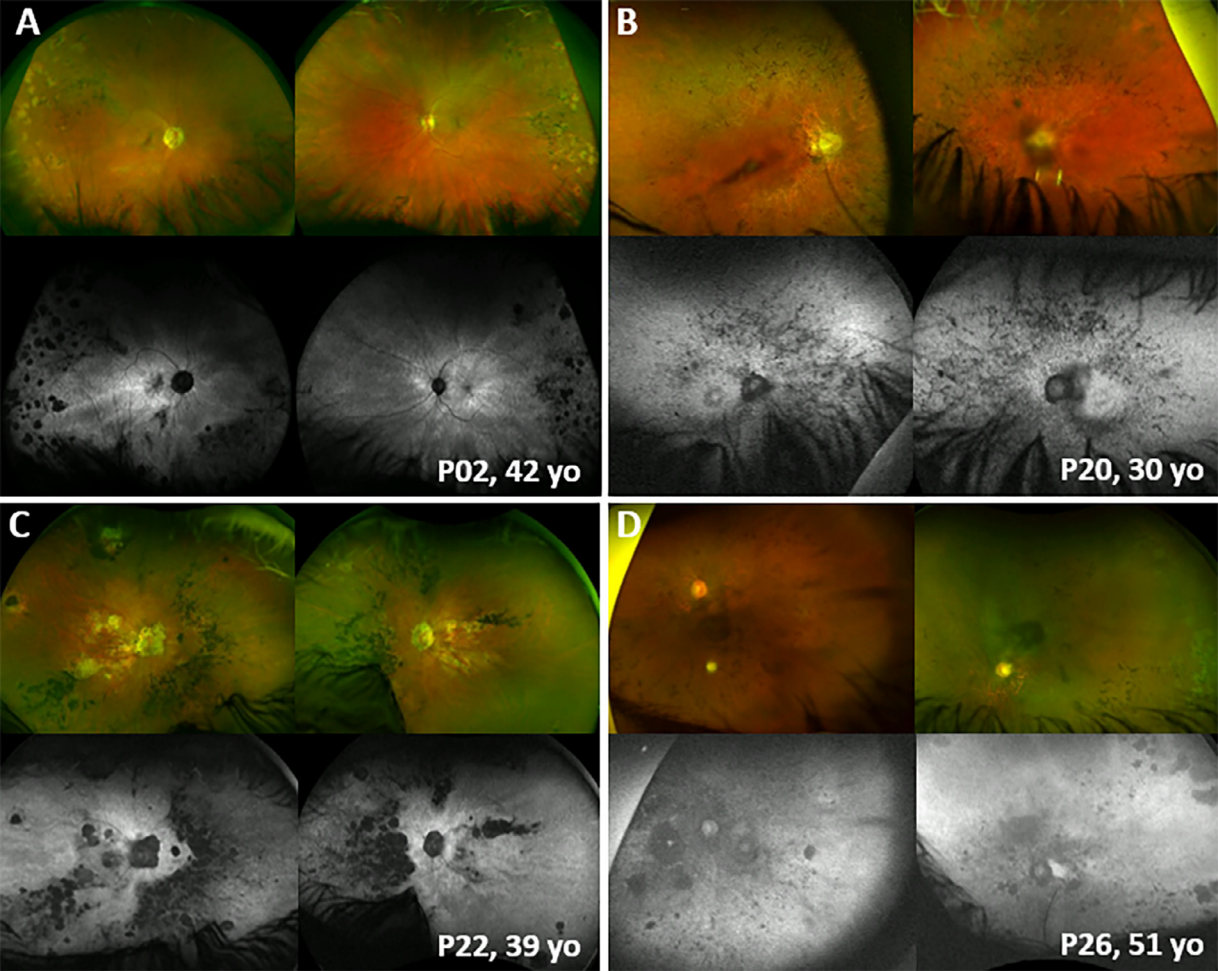
Retinal imaging of symptomatic *RP2* carriers. Ultra-widefield pseudocolor fundus images and fundus autofluorescence (FAF) (Optos Ultra, Optos, Scotland, UK) of 4 female patients with *RP2*-associated retinitis pigmentosa. Disease presented with variable severity, as well as segmental involvement. A. Greater amount of retinal degeneration temporally. B and C. Greater extent of degeneration nasally. D. Patchy areas of degeneration in all 4 quadrants. P20 had a history of retinal detachment in the right eye. P = patient; yo = years old.

Data were available only from history for 8 pedigrees, with patients reporting affected female relatives with RP in 4 cases and unaffected female relatives in the other 4 cases. Twenty-seven females from 21 pedigrees were examined by a retinal genetics specialist. Twenty-three patients (85%) reported no complaints and had normal vision; 4 patients had RP-associated complaints (15%). Eight patients had normal fundus examination (30%), 10 had a TLR (37%) (Figure 1), 5 had scattered peripheral pigmentation (19%), and the 4 symptomatic patients had fundus findings compatible with RP (15%) (Figure 2). The mean age, age range, and standard deviation for each phenotypic group at the time of the evaluation are shown in Table 1.

**TABLE 1:** Clinical Presentation (n = 27)

Complaints	n (%)	Mean Age (Range), ±SD (y)
No complaints - normal VA	23 (85.2)	42 (16-76), ±13
Nyctalopia	4 (14.8)	41 (30-51), ±7
Fundus appearance		
Normal fundus	8 (29.6)	36 (16-49), ±11
Tapetal-like reflex	10 (37.0)	41 (30-55), ±7
Scattered pigmentation	5 (18.5)	57 (41-76), ±13
RP changes	4 (14.8)	41 (30-51), ±7
Disease severity		
No disease	23 (85.2)	42 (16-76), ±13
Mild	2 (7.4)	42 and 46
Severe	2 (7.4)	41 and 51
Visual impairment^a^		
No or mild	24 (88.9)	42 (16-76), ±13
Moderate	1 (3.7)	46
Blindness	2 (7.4)	41 and 51

RP = retinitis pigmentosa; SD = standard deviation; VA = visual acuity.

a Based on World Health Organization criteria.

### Nonocular Manifestations

Similar to previously reported affected males, no nonocular manifestations were identified. However, ascertainment bias cannot be excluded, as most patients were recruited from a stand-alone eye hospital (Moorfields Eye Hospital).

### BCVA and Disease Severity

All patients with normal fundus, TLR, and isolated areas of peripheral pigmentation were normally sighted and asymptomatic (n = 23/27, 85%). Of the 4 symptomatic individuals, symptoms were as follows: patient 2 only noticed difficulties with night vision and peripheral vision after 40 years of age and had intact VA (Figure 2, A). Patient 20 had night vision problems and myopia since childhood and was diagnosed with XLRP at 21 years of age. She had BCVA of 1 logMAR (6/60) in both eyes at 31 years of age and further deterioration to 1.8 logMAR (1/60) by 41 years of age. Patient 22 had a history of night blindness and myopia since birth, and retinal detachment repair of the right eye at 16 years of age. BCVA was 6/36 and 6/9 and further deteriorated to 1/60 and 6/19 by 46 years of age. Patient 26 was symptomatic since childhood also with nyctalopia and myopia. VA was 0.78 logMAR (6/36) and 0.18 logMAR (6/9) at 36 years of age, and significantly deteriorated to 2.28 logMAR (hand motion) and 1.98 logMAR (counting fingers) at 51 years of age.

Based on previously described clinical severity grading criteria (Supplemental Table 1), of the 4 affected patients, 2 had mild disease (patients 2 and 22) and 2 had severe disease (patients 20 and 26). Based on the WHO visual impairment criteria applied to all examined carriers, 24 carriers (89%) had no or mild visual impairment, 1 affected carrier (3.7 %) had moderate impairment, and 2 affected carriers (7.4%) were blind. In total, 3 patients (11%, or 75% of symptomatic carriers) had low vision.

### Molecular Genetics and Genotype–Phenotype Associations

Thirty-eight of the 40 pedigrees examined were recently published in an *RP2* study characterizing affected males.^17^ Table 2 details the identified variants including their predicted effect and the phenotype for each patient. From the 21 pedigrees from which carriers were examined, we identified 6 frameshift alterations (28.6%), 5 missense (23.8%), 6 nonsense (28.6%) variants, 1 splice site change, 1 whole gene deletion, and 2 smaller deletions. The 2 new variants/pedigrees are marked in Table 2. From the 4 affected individuals, the 3 with more severe disease harbored null variants, and the individual with mild disease had a missense variant (patient 20). The variants for the affected individuals were located in the ferredoxin-like domain (n = 3) and the β helix domain (n = 1). The ARL3 binding domain was the most frequently affected in the cohort and none of the carriers of ARL3 binding domain variants were affected. TLR was observed in carriers with variants in all the protein domains except the myristylation/palmitoylation motif, where the only included carrier had a normal fundus.

**TABLE 2:** Genetics and Phenotype

Patient No.	Pedigree	Variant		Exon	Protein Domain	Predicted Effect	Fundus Appearance
		cDNA Change	Protein Change				
1	17177	19A>T	Lys7*	EXON 1	Myristylation	Loss of function	Normal fundus
2	26491	159_160insAA	Pro54Asnfs*5	EXON 2	β helix domain	Truncation/loss of function	Retinitis pigmentosa
3	26582	181C>T	p.(Gln61*)	EXON 2	β helix domain	Truncation/loss of function	Tapetal-like reflex
4	4488	235delG	Ala79fs	EXON 2	Cofactor C-like domain	Loss of function	Tapetal-like reflex
5	26279	256T>C	p.(Cys86Arg)	EXON 2	β helix domain	Misfolding/instability	Tapetal-like reflex
6	AR02^a^	300_301del	Phe102Profs*21	EXON 2	Arl3 binding domain	Truncation/loss of function	Tapetal-like reflex
7	20023	338C>A	Ala113Asp	EXON 2	Arl3 binding domain	Protein alternation	Tapetal-like reflex
8	15222	341G>A	Cys114Tyr	EXON 2	Arl3 binding domain	Protein alternation	Tapetal-like reflex
9	15222	341G>A	Cys114Tyr	EXON 2	Arl3 binding domain	Protein alternation	Normal fundus
10	15222	341G>A	Cys114Tyr	EXON 2	Arl3 binding domain	Protein alternation	Normal fundus
11	15222	341G>A	Cys114Tyr	EXON 2	Arl3 binding domain	Protein alternation	Peripheral pigmentation
12	20948	352C>T	Arg118Cys	EXON 2	Arl3 binding domain	Protein alternation	Peripheral pigmentation
13	20948	352C>T	Arg118Cys	EXON 2	Arl3 binding domain	Protein alternation	Tapetal-like reflex
14	15430	353 G>A	Arg118His	EXON 2	Arl3 binding domain	Protein alternation	Normal fundus
15	15430	353 G>A	Arg118His	EXON 2	Arl3 binding domain	Protein alternation	Normal fundus
16	17759	358C>T	Arg120Ter	EXON 2	Arl3 binding domain	Loss of function	Tapetal-like reflex
17	18099	460G>T	Glu154Ter	EXON 2	Arl3 binding domain	Loss of function	Normal fundus
18	34	460G>T	Glu154Ter	EXON 2	Arl3 binding domain	Loss of function	Peripheral pigmentation
19	49	568_569delindG	Pro190GlufsTer48	EXON 2	Arl3 binding domain	Loss of function	Normal fundus
20	28010	586C>T	Gln196Ter	EXON 2	Ferredoxin-like domain	Loss of function	Retinitis pigmentosa
21	5284	685-691del7	Gln229fs	EXON 2	Ferredoxin-like domain	Loss of function	Peripheral pigmentation
22	22576^a^	852delA	Ala285HisfsTer8	Exon 3	Ferredoxin-like domain	Loss of function	Retinitis pigmentosa
23	16814	969+3A>T	Splice site mutation	EXON 4/5	Ferredoxin-like domain	Protein instability	Normal fundus
24	16814	969+3A>T	Splice site mutation	EXON 4/5	Ferredoxin-like domain	Protein instability	Peripheral pigmentation
25	24452	Exon 5 deletion	NA	EXON 5	Ferredoxin-like domain	Protein instability	Tapetal-like reflex
26	4300	Exon 5 deletion	NA	EXON 5	Ferredoxin-like domain	Protein instability	Retinitis pigmentosa
27	21172	Whole gene deletion	NA	EXON 1-5	Gene deletion	Gene deletion	Tapetal-like reflex

a Pedigrees and variants not previously reported.

### FAF and OCT

FAF imaging was available for 7 patients with TLR (Figure 1) and for the 4 patients with RP (Figure 2). All the patients with clinically observed TLR had evident radial FAF changes. The patients with RP showed variable degrees of midperipheral retinal degeneration in an asymmetric pattern compared with male patients with *RP2*.^17^ Two patients with TLR had longitudinal data without progressive changes of FAF over 1 and 4.5 years, respectively. Three patients with RP had longitudinal data over 6.7, 7.6, and 11.4 years, showing slowly progressive atrophic changes over time.

OCT was available for 3 patients with RP (41, 39, and 51 years of age) and 7 patients with TLR (mean age 42 years [range 34-55 years]). Patients with RP showed atrophic and myopic changes with ellipsoid zone loss. Patients with TLR had intact retinal layers and increased reflectivity of the RPE and EZ complex (Figure 1). Two patients with RP had longitudinal data with a follow-up of 7.6 and 3.9 years with progressive atrophic changes, difficult to track because of advanced degeneration. One patient with TLR had 1 year of follow-up with stable OCT findings.

### Electrophysiology

**FIGURE 3:**
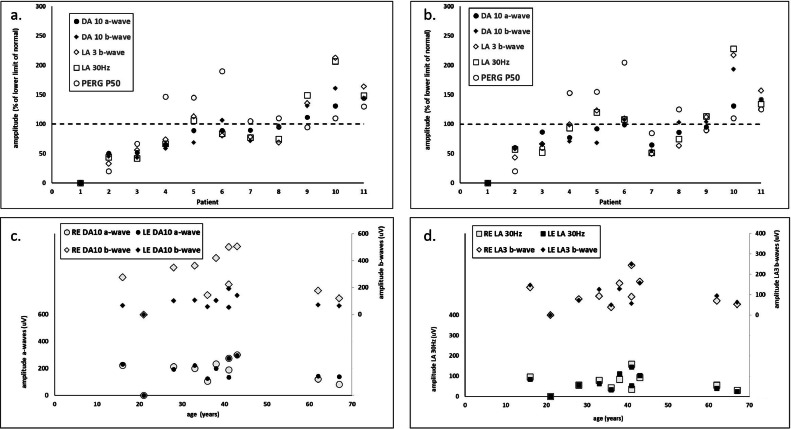
**Electroretinography (ERG) graphs.** Right eye (A) and left eye (B) full-field and pattern ERG findings summarized in 11 subjects tested according to International Society for Clinical Electrophysiology of Vision standard methods. The amplitudes of the DA10 ERG a-waves, b-waves, LA 30-Hz ERGs, LA 3 ERG b-waves, and PERG P50 components are plotted against the primary axis as a percentage of the age-matched lower limit of the (“normal”) reference range (horizontal broken line; 100%), with values arranged in ascending order of right eye DA10 ERG a-wave amplitude for clarity. The DA 10 ERG a-waves (primary ordinate axis) and b-waves (secondary ordinate axis) are compared with age for both eyes (C). The LA 30-Hz ERG (primary ordinate axis) and LA3 ERG b-waves are compared with age for both eyes (D). See text for details.

Full field ERGs were available in 11 subjects (median age 38 years [range 21-67 years]) and were quantified (Figure 3). The ERGs ranged from undetectable (n = 1; age 21 years) to bilaterally normal (n = 2; ages 41 and 43 years). One individual had mildly subnormal DA ERGs in keeping with bilateral selective loss of rod photoreceptor function (case 5 in Figure 3, A and B); others showed mild to severe attenuation of DA and LA ERGs, mostly in keeping with similar relative involvement of rod and cone systems (Figure 3, A and B). There was additional bilateral LA 30-Hz peak time delay in the 3 individuals with the smallest detectable DA10 ERG a-waves (delays 4.5-7 ms; subjects 2, 3, and 4; Figure 3) and in the left eye of 1 other (subject 7; Figure 3; delay 2 ms). Interocular ERG amplitude asymmetries (>20%; maximum 41%) were evident in a high proportion of cases (Figure 3, C and D) involving the DA 10 ERG a-waves (n = 2) and b-waves (n = 4) and LA 30-Hz (n = 5) and LA3 ERG b-waves (n = 4). Only 1 case showed a significant interocular ERG asymmetry in the LA 30-Hz ERG peak time (unilateral delay of 3 ms; subject 7). Although relatively few in number, linear regression of interocular ERG amplitude asymmetry against age revealed weak positive correlation coefficients for the DA 10 ERG a- and b-waves (r^2^ = 0.44 and 0.52, respectively; *P* < .05), and no significant correlation for the LA 30-Hz ERG and LA3 ERGs.

Pattern ERG P50 components were normal bilaterally in 6 subjects. In the others, P50 was bilaterally undetectable (n = 1) or subnormal (n = 2) in the 3 subjects with most severe DA 10 ERG a-wave reductions, or were marginally subnormal (3 eyes of 2 subjects including the left eye of case 7 in Figure 3). Interocular PERG P50 amplitude differences were <12% in all but 1 case (subject 7 in Figure 3; interocular difference 19%).

## Discussion

This study details the clinical phenotype of *RP2* retinopathy in a large cohort of females. Most carriers were asymptomatic, exhibiting subclinical characteristics such as TLR and pigmentary changes, with only 4 carriers of *RP2* variants manifesting RP. Detailed electroretinography in a cohort of 11 revealed a wide range of retinal function phenotypes, including interocular asymmetries.

In contrast with some other forms of progressive inherited retinal diseases,^30^^,^^31^ similar to affected males, there was less dissociation of structure and symptoms; symptomatic patients had more severe degeneration and asymptomatic carriers had subtle peripheral changes, TLR, or normal fundus. The disease spectrum is likely explained by Lyonization, whereby random X-chromosome inactivation during embryogenesis leads to variable expression of the wild-type phenotype. Nevertheless, further genotype–phenotype correlations cannot be excluded; the 3 more severely affected carriers had null variants in the ferredoxin-like domain. However, other carriers with C-terminal null variants also exhibited normal fundus, pigmentary changes, or TLR.

Examination of female carriers can facilitate the diagnosis of XLRP given the high frequency of clinical findings. Family history of affected females with RP does not exclude X-linked disease. No notable age difference was observed between affected and unaffected individuals, as well as among patients with different disease severity. In the current study, 4 pedigrees out of the 40 screened (10%) had affected-symptomatic females. Comander and associates^8^ reported that 2% of XLRP carriers (in a cohort of *RPGR, RP2*, and not molecularly confirmed carriers) were blind; applying the WHO criteria for visual impairment in the current cohort, 7.4% were blind (n = 2). The discrepancy in the percentage likely reflects the smaller size of our cohort, or the fact that in the former study only 6 pedigrees with molecularly confirmed *RP2* disease were included. The affected carriers in the current study had a diverse presentation ranging from late-onset mild RP to early-onset severe degeneration. We have reported a similar phenotypic spectrum for *RPGR*-affected carriers, ranging from sector RP to severe early-onset retinal degeneration.^32^^,^^33^ Saeed and associates,^34^ in a recent meta-analysis of 13 studies, had similar conclusions to ours, including preservation of good BCVA for most female carriers, variable phenotype, and greater BCVA loss for affected males.

Full-field ERGs were abnormal in 9 of 11 cases, revealing rod and cone photoreceptor dysfunction of widely differing severity. Unlike most retinal dystrophies, there was a high degree of interocular ERG asymmetry, consistent with previous studies on other cohorts of obligate carriers of XLRP.^7^ It is interesting to note that although there were relatively few subjects, interocular ERG asymmetry in the rod-mediated ERG components correlated with age, suggestive of asymmetrical or unilateral progression, and broadly consistent with a higher incidence of ERG abnormality in obligate carriers of older age.^8^ Those with mild retinal dysfunction had PERG evidence of normal or relatively preserved macular function. The PERG was undetectable in only 1 case, associated with undetectable full-field ERGs and a severe loss of photoreceptor function. Patients 5, 6, 7, and 10 (Figure 3), despite having documented normal fundus, had variable ERG findings, with only patient 10 having ERG within normal limits.

Future prospective studies with standardized imaging acquisition protocols will acquire multimodal data in all patients, as well as longitudinal data. The use of novel high-resolution imaging techniques such as adaptive optics scanning laser ophthalmoscopy may further help to clarify the cellular basis of TLR and pathogenesis of disease.^35^^,^^36^ The retrospective nature of the current study has inherent limitations. Imaging data, cross-sectional and longitudinal, were not available for most of the patients, and the functional assesments did not include visual field or dark adaptation testing.

This study details the clinical phenotype of *RP2* retinopathy in a large cohort of females. Most carriers were asymptomatic, exhibiting subclinical characteristics such as TLR and pigmentary changes. However, female carriers of *RP2* variants can manifest RP. The phenotypic spectrum as described herein has prognostic and counselling implications for *RP2* carriers and patients.

## Appendix Supplementary materials

Image, application 1
