# Supplementary material for: RP2-Associated X-linked Retinopathy: Clinical Findings, Molecular Genetics, and Natural History in a Large Cohort of Female Carriers
Source: Am J Ophthalmol. 2024 May;261:112–20. doi: 10.1016/j.ajo.2023.11.005 (PMC11139645; doi:10.1016/j.ajo.2023.11.005)
Supplement: Supplementary file 1 [file mmc1.docx]

# Supplementary Table 1: Visual Acuity Grading Clinical Severity Grading*

**Mild Disease**

BCVA greater than 0.40 LogMAR (20/50)

**Less severe**

Late onset (>30 years) and VA worse than 0.40 LogMAR (20/50)

| **Severe** |  | |
| --- | --- | --- |
| BCVA worse than: |  | at age (years): |
| 0.40 LogMAR (20/50) |  | <20 |
| 0.70 LogMAR (20/100) |  | 21-30 |
| 1.00 LogMAR (20/200) |  | 31-40 |
| 1.30 LogMAR (20/400) |  | >41 |
|  |  |  |
| **WHO Visual Impairment Criteria** |  |  |
| No or Mild Visual Impairment | < 0.48 | LogMAR |
| Moderate Impairment | 0.48 -1 | LogMAR |
| Severe Impairment | 1-1.3 | LogMAR |
| Blindness | >1.3 | LogMAR |
| *Criteria adjusted from *Jayasundera et al.* |  |  |
